# Bilateral Postoperative Cyst after Maxillary Sinus Surgery: Report of a Case and Systematic Review of the Literature

**DOI:** 10.1155/2016/6263248

**Published:** 2016-07-11

**Authors:** Boris-Mark Niederquell, Peter A. Brennan, Michael Dau, Maximilian Moergel, Bernhard Frerich, Peer Wolfgang Kämmerer

**Affiliations:** ^1^Department of Oral, Maxillofacial and Plastic Surgery, University Medical Centre Rostock, Schillingallee 35, 18057 Rostock, Germany; ^2^Maxillofacial Unit, Queen Alexandra Hospital, Southwick Hill Road, Cosham, Portsmouth, Hampshire PO6 3LY, UK; ^3^Department of Oral and Maxillofacial Surgery-Plastic Surgery, University Medical Centre Mainz, Johannes Gutenberg University, Augustusplatz 2, 55131 Mainz, Germany

## Abstract

*Purpose*. We present a case of a bilateral postoperative maxillary cyst (PMC) and discuss this with a systemic review.* Case Report and Literature Review*. A 68-year-old female with pain and swelling on the right side of the face. MRI and CT showed a cystic tumors of the right and left maxillary sinus. Radical maxillary surgery via a Caldwell-Luc procedure had been performed 55 years ago and bilateral PMC was diagnosed. The PubMed database was searched for PMC within the last 30 years.* Results*. Together with the current case, we found 23 reports including 284 patients describing PMC. It was diagnosed at a mean time of 22 years after causal surgery at a mean age of 47 years. Initial symptoms were mostly pain with or without swelling. The main radiological sign was a unilocular radiolucency with a slight preference for the left side.* Discussion*. PMC is a long-term complication that can occur after maxillary sinus surgery and a second surgical approach is required in order to stop cystic expansion. Therefore, patients' informed consent on this complication as well as a prolonged follow-up is recommended. Simple paranasal ultrasound or paranasal sinus plain radiography may lead to an earlier detection reducing interventional morbidity.

## 1. Introduction

Postoperative maxillary cysts (PMCs) may occur as a delayed complication up to 60 years after maxillary sinus surgery [1–4]. The incidence of PMCs depends on aetiology and they are more often found in Japan than in the Western world [5]. Their occurrence following Caldwell-Luc operations is 1 : 150 and PMC account for 19.5% of all cystic lesions of the jaws. PMC may originate from obliteration of the sinus ostia as well as from residual mucosa [1, 6].

Symptoms of PMC are swelling or pain in the buccal or mucogingival fold region of the maxilla, discomfort of the maxilla or maxillary teeth, and exophthalmus. Characteristic radiographic findings are unilocular or multilocular cystic radiolucency with a well-defined margin and surrounding sclerotic bone in the floor of the maxillary sinus, with or without bony perforation [7, 8]. The biochemical characteristics of the fluids demonstrate a transudate rather than exudate [9]. Histopathologically, cuboidal, squamous, and mixed epithelial cyst linings were observed; although the basic epithelial lining was the ciliated columnar type, epithelial dysplasia is possible [1]. It is obvious that a late finding of PMC is often combined with an increase in discomfort or pain. Advanced PMCs can erode bony sinus walls due to higher intraluminal pressure. In these cases, herniation of nearby structures such as orbital tissues or the overlying skin could occur as well. Early detection is advisable. We present a case of late, bilateral PMC. The incidence of PMC after maxillary sinus surgery and the latency of PMC occurrence are evaluated in a systematic review.

## 2. Case Presentation

**Figure 1:**
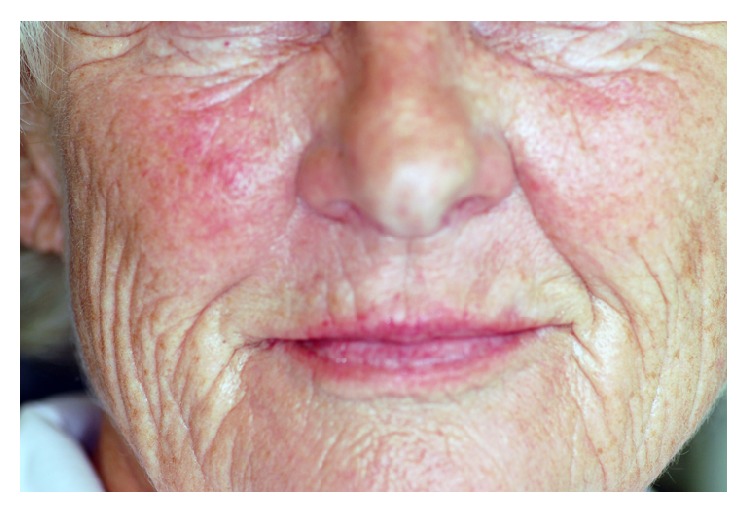
Clinical findings: discreet swelling and reddening infraorbital on the right side, only visible in comparison to the contralateral side.

**Figure 2:**
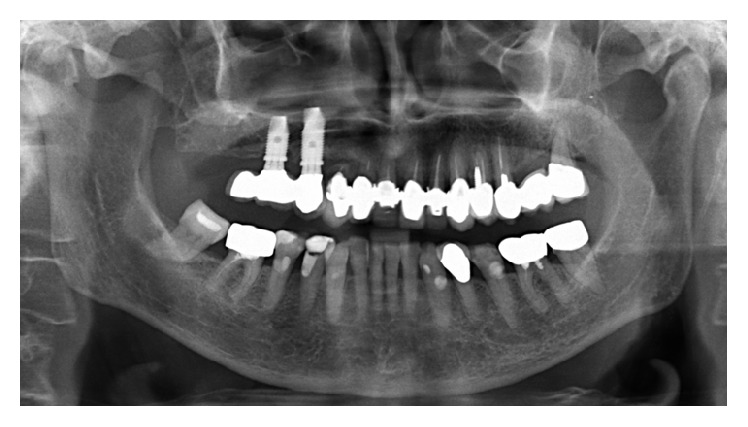
Panoramic X-ray: unilocular, well-defined radiolucency of the right maxillary sinus region without dental focus.

**Figure 3:**
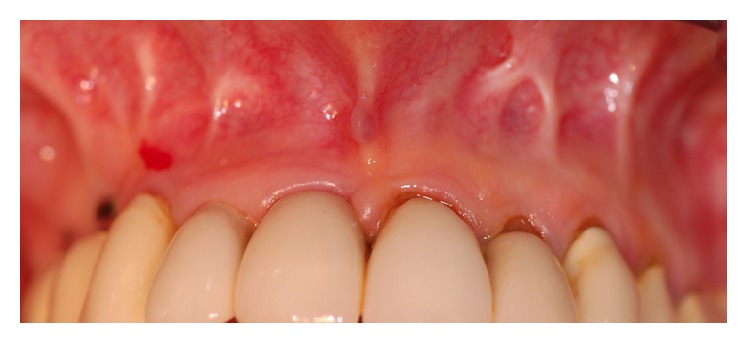
Oral view: scarred tissue in the vestibule of the upper jaw after surgery in childhood.

**Figure 4:**
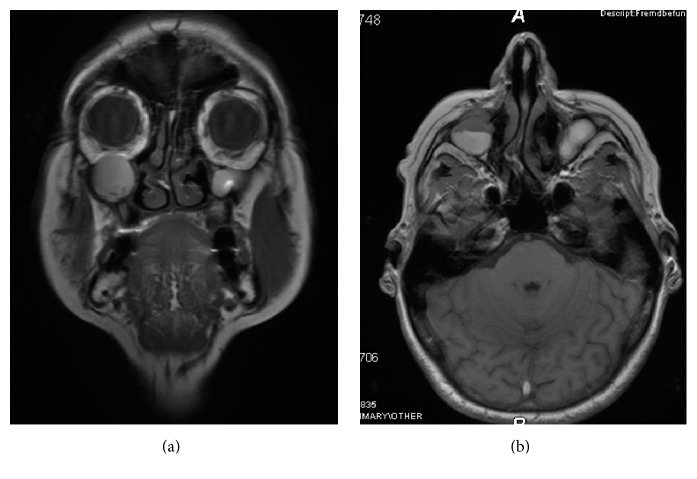
MRT (T2): coronal (a) and sagittal (b) view. Bilateral cystic lesions of the maxillary sinus with elevation of the orbital floor on the right side.

**Figure 5:**
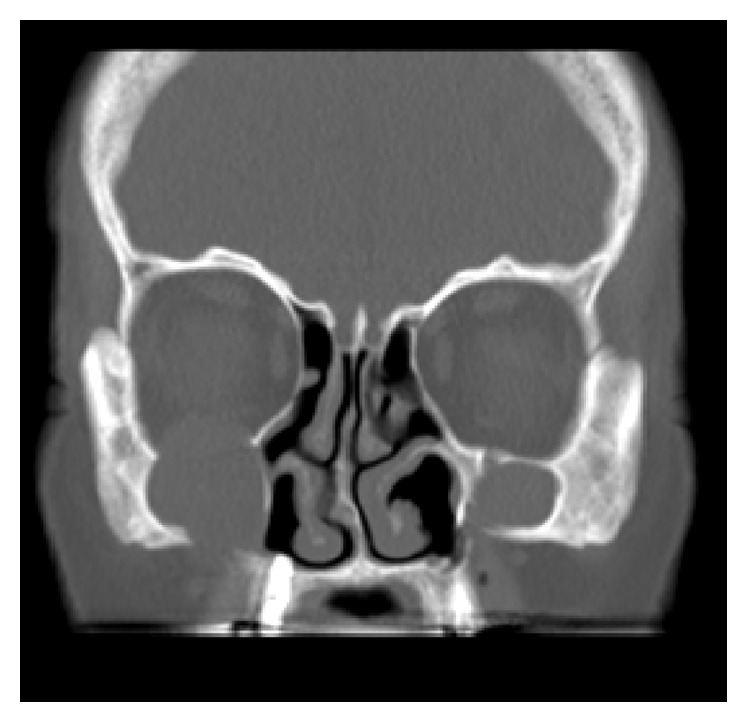
CT: coronal view showing osseous defects in the nasal part of the right sinus and the orbital floor.

**Figure 6:**
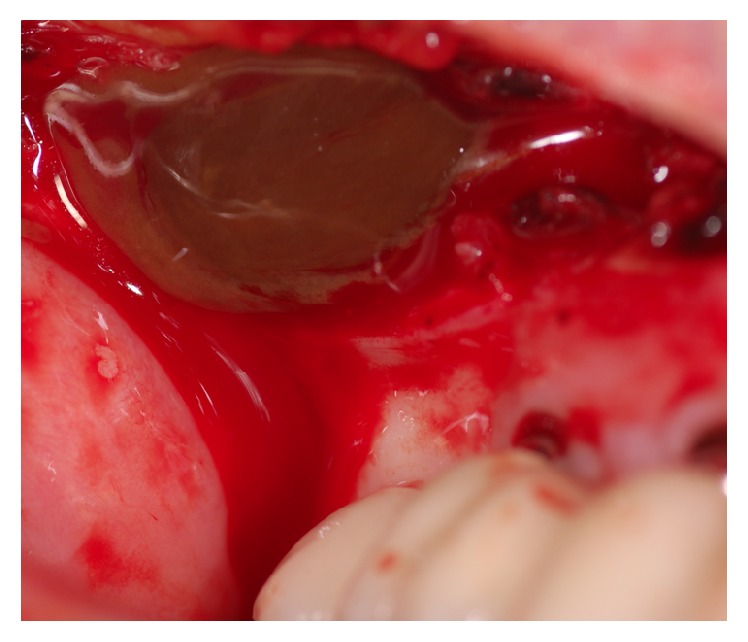
Intraoperative view of the right maxillary sinus: after incision and preparation of the right upper jaw, a defect of the facial bone sinus on the right side appears.

**Figure 7:**
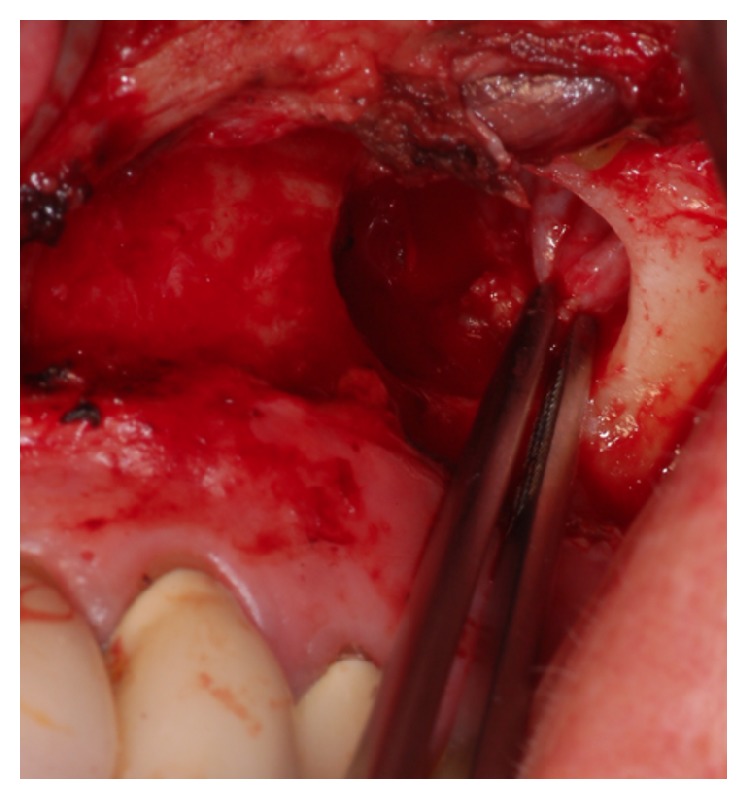
Intraoperative view of the left maxillary sinus: after preparation on the lift side, a bony defect is visible, smaller than on the right side. After osteoclastic preparation, the whole cystic lesion was removed.

**Figure 8:**
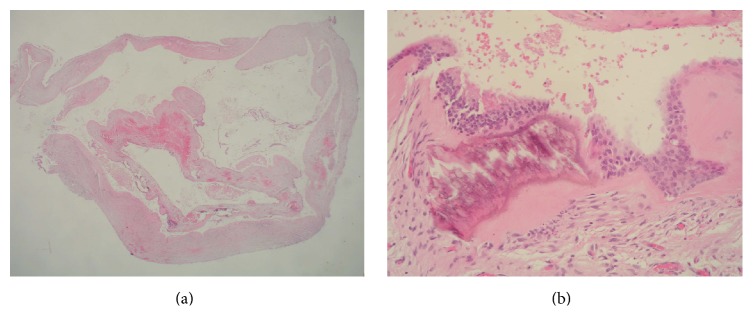
(a) Histology: overview of the cystic tissue with focally intact epithelium and loose fibrotic stroma (haematoxylin and eosin; original magnification ×20). (b) Histology: cystic wall with thin, fibrotic, and partly edematous tissue. Little inflammatory infiltrate with granulocytes; cyst lined by a combination of stratified nonkeratinizing squamous epithelium and pseudostratified ciliated columnar epithelium (haematoxylin and eosin; original magnification ×20).

A 68-year-old female consulted the Department of Oral, Maxillofacial and Plastic Surgery with a painful swelling on the right infraorbital side of the face (Figure 1). Intraorally, no signs were noted (Figure 2), apart from scars in the region of the unattached gingiva of the maxilla (Figure 3). The patient underwent a Caldwell-Luc operation at the age of 13 on both sinuses due to chronic maxillary sinusitis. She had a mastectomy for breast cancer following irradiation 31 years previously. MRI and CT found a cystic lesion of the right maxillary sinus with orbital floor elevation. In the left maxillary sinus, a cyst was detected as well (Figure 4). Bony defects were visible in the nasal part of the right sinus and the orbital floor (Figure 5). At operation, after incision of the gingiva, no bony wall of the right maxillary sinus was evident (Figure 6). The cystic tissue was adherent to the buccal soft tissue and the infraorbital nerve. Cystectomy was performed with infraorbital nerve preservation and cystic tissue was removed intact (Figure 7). A sinuscopy revealed minor damage of the orbital floor; the medial sinus wall was missing after Caldwell-Luc surgery. No further reconstruction was necessary. A pack was inserted in the inferior nasal meatus for 5 days. On the left side, cystic tissue was removed in the same way as described above and a pack in the inferior meatus was removed after 3 days. Clinical signs and histological report confirmed a postoperative bilateral maxillary cyst (Figures 8(a) and 8(b)). No diplopic images were stated but the patient complained about hypoesthesia of the right infraorbital nerve. After half a year, CT control showed no recurrence. Sensory disturbances disappeared during the follow-up examinations within one year after removal of the cysts.

## 3. Review of the Literature

### 3.1. Search Strategy

A PubMed database literature search was performed to analyze the etiology, incidence, and latency of postoperative maxillary cysts (PMCs). Search strategy contained the keywords “postoperative maxillary cyst”, “surgical ciliated cyst”, “postoperative paranasal cyst”, and “respiratory implantation cyst”. Results were limited to English and German literature of the last 30 years. Out of the included articles, patients' age, gender, initial symptoms, location, kind of initial surgery, time after initial surgery, radiographic features, size, histopathology, possible recurrences, and length of follow-up examinations were extracted.

### 3.2. Results of the Literature Review

PMC is a rare complication after maxillary sinus surgery. Together with the current report, a total of 23 articles describing 284 patients with PMC were found (Table 1). The mean age was 47 years (min: 19, max: 85); male patients (57%) were affected slightly more often than females (43%). Complained symptoms were unspecific such as pain (50%), pain and swelling (19%), swelling only (5%), sight disorder (2%), and tenderness (2%). In 23% of cases, symptoms were not available. Unilateral incidence was described more often (32%) than bilateral (10%). Slightly more often cysts were on the left (20%) side than on the right side (13%) whereas in the majority of cases (67%) no respective data were given. In 67% of cases Caldwell-Luc was the initial surgery, 2% underwent Le Fort osteotomy, and 31% had no specifications or other surgeries. The average time of latency for PMC after first surgery was 22 years (min: 0,5, max: 60).

## 4. Discussion

Postoperative occurring maxillary cysts (PMCs), which are also known as mucoceles, surgical ciliated cysts, postoperative paranasal cysts, or respiratory implantation cysts, are benign lesions of the maxillary sinuses [2, 10]. They are long-term complications after surgery associated with maxillary sinuses [2, 3]. In accordance, this case report showed occurrence of bilateral PMC 55 years after ablative maxillary surgery.

PMC was firstly described by Kubo in 1927, who proposed two hypotheses to explain the pathogenesis, trapped mucosa from a former surgical treatment or retention of blood or tissue fluid forming a space after surgery [28]. Yamamoto and Takagi suggested that cysts that are located far from the nasal cavity arise from entrapped mucosa after surgical treatment and cysts that are near the nasal cavity arise from regenerated mucosa or from nasal mucosa [1]. More commonly reported in Asians than Caucasians, the explanation for the different incidence remains unclear. Nishioka et al. suggested a significantly higher prevalence in Japan, compared to other countries, due to different infectious agents and bone structure. His workgroup found in a 20-year literature review an incidence of PMC in Asian 1223 cases versus 87 cases in Caucasian [29]. Basu et al. proposes that PMC is not as rare as described in former studies; improper diagnosis should lead to this incidence [24]. Late diagnosis of PMCs could result in eroded bone and pressure resorption together with herniation of surrounding structures. Fewer radical maxillary surgery is performed today, like Caldwell-Luc, as other less traumatic approaches are used. To prevent PMCs, minimal invasive surgery of maxillary sinuses seems to be of high importance. Though some have found that PMCs occurred after less invasive sinus surgery as well [30]. Reliable data of minimal surgery will only be available in retrospective view [3]. Avoidance of maxillary sinus surgery, if possible, could be advantageous.

If sinus surgery is needed, details about the risks and complications and the possibility of occurring PMCs before every surgical treatment of the maxillary sinus should be discussed with patients. Due to the long latency, a radiological long-term follow-up for patients who underwent surgical treatment of the maxillary sinus could be favorable.

## Figures and Tables

**Table 1:** Overview of clinicopathological features of PMCs obtained from the literature.

Author	Year	Patient (age, gender)	Initial symptoms	Location maxillary sinus	Radiographic features	Initial surgery (cause)	Time after initial surgery (years)
[10]	2014	47 m	Pain, swelling, pressure	R & L	n.a.	CL	28
		65 f		R		CL	50
		35 f		L		CL	10
		61 f		R		CL	23

[11]	2013	60 m	Swelling	R	Unilocular radiolucency	Augmentation	11

[12]	2012	45 f	Swelling and pain	L	Radiolucent lesion	CL	2
		28 m		R		CL	13
		49 f		L		CL	26

[2]	2010	54 f	Repeated swelling	L	Unilocular radiolucency; expansion into surrounding soft tissue	“A maxillary sinus surgery”	8

[4]	2009	60 f	n.a.	R	Smooth & lobulated borders	n.a.	n.a.
		58 m	n.a.	R		n.a.	41
		44 f	n.a.	L		n.a.	22
		75 f	n.a.	R		n.a.	60
		59 m	n.a.	R & L		n.a.	15
		75 f	n.a.	L		n.a.	55
		64 m	n.a.	L		n.a.	40

[13]	2009	18 males, 20 females (mean 46,5 years)	n.a.	n.a.	Completely opacified maxillary sinus with evidence of expansion	CL	21

[14]	2009	56 f	Discomfort	R	Unilocular, translucent area with radiodense borders	CL	3

[15]	2009	35 f	Swelling	Midline of the palate	Well-defined cyst	Le Fort I osteotomy	7

[16]	2003	32 m	Chronic dull pain and tenderness	L	Extensive unilocular cystic lesion related to the left maxilla and causing marked expansion and thinning of the surrounding bone	Le Fort I osteotomy	15

[17]	2003	31 f	pain	L	Opacification of the left maxillary sinus	Le Fort I osteotomy	15

[18]	2000	28 m	n.a.	n.a.	n.a.	CL	4
		72 m					20
		57 f					25

[19]	2000	41 f	None	L	Round, well-defined cystic cavity	Maxillary sinus augmentation	0.5

[20]	1993	15 males, 9 females (mean 47,4 years)	Most had pain and extraoral swelling	n.a.	Unilocular cysts	Maxillary surgical intervention	8–55

[21]	1993	50 m	Exophthalmos and diplopia	L	Mucocele protruding into the left orbit	CL	31
		61 f		L		CL	43

[22]	1990	39 f	Pain, swelling, pus	L	(I) n.a.	(I) Le Fort III osteotomy	4
		21 m		L	(II) Cystic lesion	(II) Le Fort II osteotomy	3/5
		38 m		L	(III) Cystic lesion	(III) Le Fort I osteotomy	3

[23]	1990	41 m	Swelling, pain, pus, tenderness, discomfort	R & L	13 cysts were involved the sinus completely, 12 were multilocular, 13 exhibited incomplete septa	Operations for maxillary sinusitis	21
		47 f		L			27
		33 m		R & L			15
		52 m		R & L			32
		40 f		R & L			25
		37 m		R & L			22
		45 f		R & L			24
		57 m		R			18
		68 m		R & L			43
		36 m		R & L			14
		63 m		L			25
		44 m		R & L			26
		57 m		R			40
		52 m		L			15
		37 m		R & L			22
		50 m		R & L			26
		36 f		R			23
		63 m		L			27
		54 m		L			10
		38 f		L			24
		49 f		R & L			25
		67 m		R			48

[24]	1988	9 males and 12 females 25 to 74 years (mean of 43.7 years)	Tenderness, pain, swelling	19x unilateral 2x bilateral	Cystic lesions	Antral surgery	7–39 (mean 20)

[25]	1988	21 m	Swelling and pain	R	Large radiolucent area	Apicoectomies of the buccal roots	1/2

[26]	1987	28 m	Tenderness, pain, swelling, facial distortion, diplopia	R	Lucent, expansive lesion with dehiscence in the bony wall	CL bilateral	14
		85 m		R		CL right	50
		57 f		R		Rights sinus surgery	49
		33 m		R		n.a.	n.a.
		65 m		R		CL right	49

[7]	1980	66 f	Swelling and tenderness	L	Small oval shaped radiolucency with a well-defined margin and with surrounding sclerotic bone in the right maxillary sinus	CL bilateral	40
		33 f		L		CL bilateral	19

[5]	1979	80 males, 52 females, 11–71 years	Pain	n.a.	Mucocele and bony erosion	CL	12–43

[27]	1978	19 m	Swelling, tenderness, fistula	L	Cystic lesions with well-defined margins and partly irregular shape and destructive growth	Operation on maxillary sinus	6
		53 m		L			35
		36 m		R			20
		39 m		L			6
		29 f		L			15
		21 m		L			7
		36 m		L			20

m: male, f: female, R: right, L: left, n.a.: not available, and CL: Caldwell-Luc operation.
